# Supplementary figures and images for: Spatial Analysis of Dengue Clusters at Department, Municipality and Local Scales in the Southwest of Colombia, 2014–2019
Source: Trop Med Infect Dis. 2023 May 2;8(5):262. doi: 10.3390/tropicalmed8050262 (PMC10222113; doi:10.3390/tropicalmed8050262)

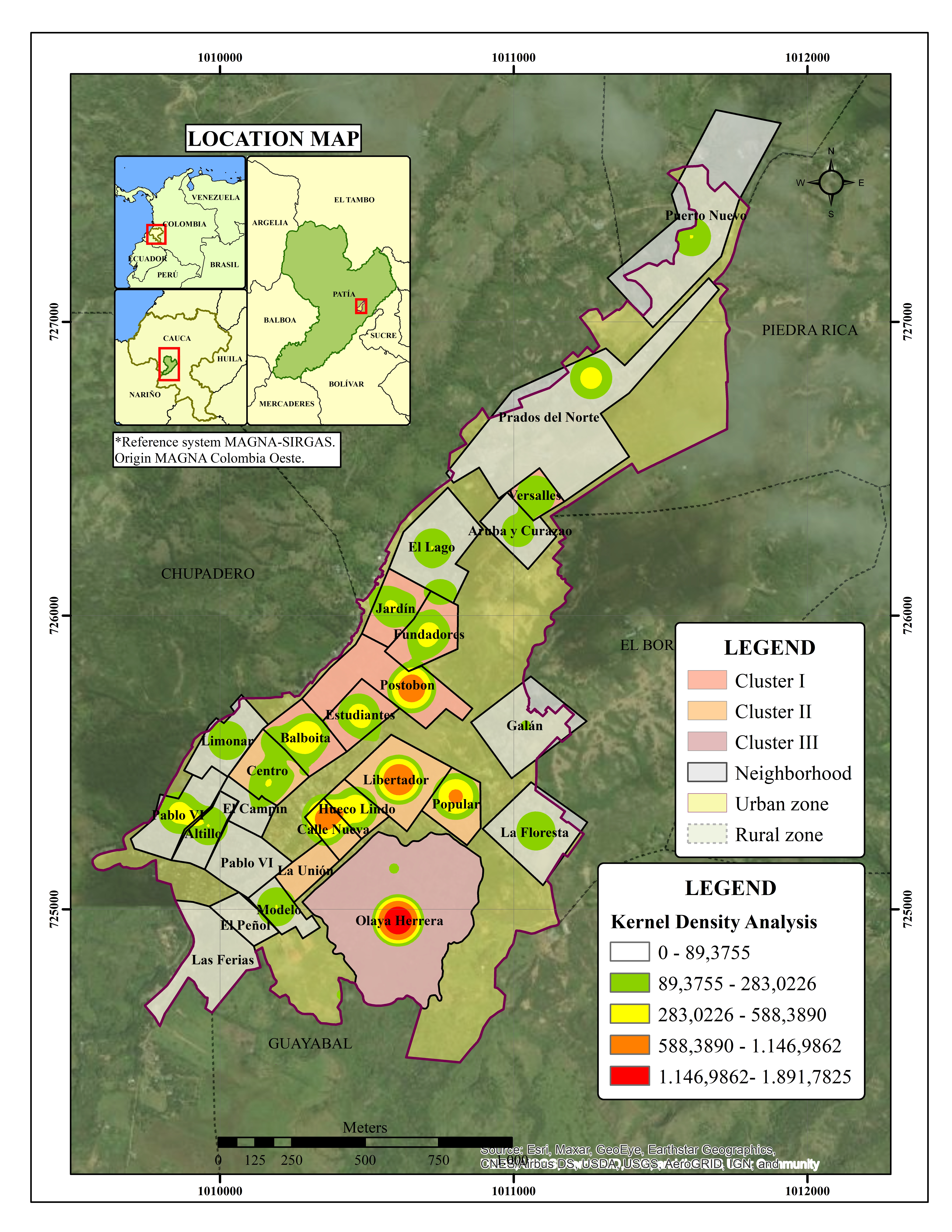

Supplement: Supplementary file 1 [file tropicalmed-08-00262-s001.zip › tropicalmed-2211392-supplementary/Figure S2.jpg]
